# The combination therapy of transarterial chemoembolisation and sorafenib is the preferred palliative treatment for advanced hepatocellular carcinoma patients: a meta-analysis

**DOI:** 10.1186/s12957-020-02017-0

**Published:** 2020-09-11

**Authors:** Zhoujing Cheng, Lin He, Yingjie Guo, Yuhua Song, Shasha Song, Lijiu Zhang

**Affiliations:** 1 Department of Gastroenterology, The Second Hospital of Anhui Medical University, No.678 Furong Road, Jingkai District, Hefei, Anhui Province China; 2 Breast Center B ward, The Affiliated Hospital of Qingdao University, Shandong Province, Qingdao, China; 3 Department of Gastroenterology, The Affiliated Hospital of Qingdao University, Shandong Province, Qingdao, China

**Keywords:** TACE, Sorafenib, Hepatocellular carcinoma, Meta-analysis

## Abstract

**Background:**

To compare the efficacy of three types of palliative therapy for advanced hepatocellular carcinoma (HCC), including transarterial chemoembolisation (TACE) monotherapy, sorafenib alone and their combination.

**Methods:**

The databases of PubMed, Embase and Cochrane Library were retrieved. The odds ratio (OR) with its 95% confidence interval (CI) was used to investigate the binary variables, and the standardised mean difference (SMD) with its 95% CI was employed to evaluate the continuous variables. All statistical tests were performed by using Stata/SE, version 12.0.

**Results:**

Thirty-one clinical studies, containing 5125 unique cases of patients with advanced HCC, were included. There were significant improvements in overall survival (OS) (pooled SMD = 2.54; 95% CI 1.74–3.34) and time to progression (TTP) (pooled SMD = 2.49; 95% CI 0.87–4.12) of the patients after receiving the combination therapy of TACE and sorafenib, compared to TACE monotherapy, and the OS in the combined treatment cohort was also longer than that in the sorafenib-alone cohort (pooled SMD = 2.92; 95% CI 1.72–4.13). The combination therapy group in comparison to the TACE group benefited a significantly increased overall response rate (ORR) (pooled OR = 2.61; 95% CI 1.43–4.77), 1-year (pooled OR = 2.96; 95% CI 1.71–5.14) and 2-year (pooled OR = 1.64; 95% CI 1.18–2.28) survival rates and reduced disease progression rate (DPR) (pooled OR = 0.47; 95% CI 0.33–0.68); in parallel, the ORR in the group was also significantly higher than that in the sorafenib-alone group (pooled OR = 3.62; 95% CI 1.28–10.22), although without a difference in the DPR (pooled OR = 0.28; 95% CI 0.05–1.48). In addition, we discovered that the 1-year (pooled OR = 1.39; 95% CI 0.84–2.29) and 2-year (pooled OR = 1.70; 95% CI 0.69–4.18) survival rates in the TACE monotherapy cohort were not significantly different to those in the sorafenib-alone cohort.

**Conclusion:**

The combination therapy is more effective than monotherapy in improving the prognostic outcomes of patients with advanced HCC. Therefore, we recommend it as the preferred treatment intervention for those patients.

## Background

Hepatocellular carcinoma (HCC) is one of the most common gastrointestinal malignancies and the third most common cause of cancer-related death, with an approximate proportion of 90% in primary malignant liver tumours in adults [1, 2]. The most effective way to treat a tumour in HCC is surgically, but only less than 18% of patients undergo it [3]; many patients are deprived of the surgery opportunity when they are initially diagnosed with an advanced-stage disease. For patients with Barcelona Clinic Liver Cancer (BCLC) stage B or C HCC who are not eligible for surgery [4], it is recommended to receive transarterial chemoembolisation (TACE) or sorafenib as the treatment modality.

The implementation of TACE is mainly constituted of two steps: (1) the embolisation of the tumour-supplying arteries to induce tumour hypoxia and necrosis and (2) the delivery of a high concentration of cytotoxic chemotherapy medications through those arteries to reinforce the tumour necrosis [5]. However, the level of vascular endothelial growth factor (VEGF) increases after TACE [6, 7], which is considered a partial facilitator of tumour progression and metastasis [8].

Sorafenib is an oral multikinase inhibitor and has the ability to inhibit tumour cell proliferation and angiogenesis [9] by suppressing the VEGF signal pathway by inhibiting VEGF receptors [10]. Some phase III, randomised, placebo-controlled trials have demonstrated its efficacy in treating advanced HCC, significantly prolonging the time to progression (TTP) and overall survival (OS) [9, 11]. Considering that sorafenib can inhibit VEGF signalling, it may be effective to reduce TACE-induced overproduction of VEGF, hence further ameliorating the disease control of advanced HCC after TACE. As expected, the results of many studies have indicated that patients with this carcinoma derived more survival benefits from the combination of sorafenib and TACE than from TACE alone [12–14].

To understand the effectiveness of TACE, sorafenib and their combination in the treatment of advanced HCC patients comprehensively, this meta-analysis, with a massive number of cases, aimed to collect all relevant data to compare the TTP, OS, disease progression rate (DPR), survival rate and overall response rate (ORR) of patients after different alleviative treatments.

## Methods

### Search strategy

The PubMed, Cochrane Library and Embase databases were electronically searched with the following retrieval strategy, in light of the Preferred Reporting Items for Systematic Review and Meta-Analysis (individual participant data) (PRISMA-IPD) statement [15]: ((“Liver Neoplasms”[MeSH]) OR (Neoplasms, Hepatic) OR (Neoplasms, Liver) OR (Liver Neoplasm) OR (Neoplasm, Liver) OR (Hepatic Neoplasms) OR (Hepatic Neoplasm) OR (Neoplasm, Hepatic) OR (Cancer of Liver) OR (Hepatocellular Cancer) OR (Cancers, Hepatocellular) OR (Hepatocellular Cancers) OR (Hepatic Cancer) OR (Cancer, Hepatic) OR (Cancers, Hepatic) OR (Hepatic Cancers) OR (Liver Cancer) OR (Cancer, Liver) OR (Cancers, Liver) OR (Liver Cancers) OR (Cancer of the Liver) OR (Cancer, Hepatocellular) OR ((Liver OR Hepatic OR Hepatocellular) AND (Tumour OR Cancer OR Tumour OR Carcinoma OR Neoplasm)) OR (Cholangiocellular carcinoma) OR Cholangiocarcinoma OR HCC-CC OR (combined HCC-CC) OR CHC OR (Mixed hepatocellular and cholangiocarcinoma)) AND ((TACE OR (Transcatheter arterial chemoembolisation) OR (Transcatheter hepatic arterial chemoembolisation) OR (Transarterial chemoembolisation)) AND Sorafenib) AND (Survival OR Response OR ORR OR OS OR (Overall survival) OR TTP OR (Time-to-progression) OR Progression). There were no restrictions during the retrieval process. The due date of citation searching was April 20, 2019.

### Inclusion criteria

- Clinical trials published in English;
- Patients with advanced HCC;
- Publication recorded the prognoses of at least two treatment methods; and
- The prognoses at least included more than one of the following components: OS, TTP, ORR, DPR, 1-year survival rate and 2-year survival rate. OS referred to the duration from the date of diagnosis to the date of death or lost to follow-up. TTP was defined as the time from randomisation to the appearance of radiologic evidence of disease progression. ORR was evaluated by enhanced computed tomography or magnetic resonance imaging before and after treatment. The assessment criteria of tumour progression and tumour response were both according to Response Evaluation Criteria in Solid Tumours (RECIST) version 1.1 or modified RECIST (mRECIST).

### Exclusion criteria

- Non-English publication
- Single-arm study
- Article type: review, case report, study protocol and conference paper
- Other details that did not meet the inclusion criteria

The titles and abstracts of all citations were screened by two co-authors independently. They further respectively perused the full texts of potential studies and retained only the satisfactory ones that met the inclusion criteria. Any inconsistencies were resolved by discussion.

### Data abstraction

Two co-authors used Microsoft Excel version 2016 (Microsoft Corporation, Redmond, WA, USA) to abstract the following information from all eligible studies: first author, publication year, study type, original nation, prognostic endpoint, number of analysed cases, median follow-up, frequency of tumour assessment, median age, drugs administrated in TACE and the initial sorafenib administration. If any disagreements existed, they were resolved by the third co-author.

### Statistical analysis

The comparison of continuous variables involving TTP and OS was assessed by standardised mean difference (SMD) with its 95% confidence interval (CI). Moreover, the crude odds ratio (OR) with its 95% CI was used to evaluate the comparison of ORR, DPR and 1-year and 2-year survival rates between different treatment interventions. The heterogeneity across included studies was detected by heterogeneity chi-squared test with its significance level of *P* < 0.1 [16]. If the heterogeneity test was not statistically significant, the data was pooled by a fixed-effects inverse variance model; otherwise, a random-effects inverse variance model was used [16]. Egger’s test, with its significance level of *P* < 0.05, was used to detect the publication bias in all analyses, and a tool presented by Jadad and colleagues was applied to evaluate the quality of all randomised controlled trials (RCTs) (eTable 1 in Supplementary, page 1) [17]. All statistical tests were performed with Stata/SE software, version 12.0.

## Results

### Search results

**Fig. 1:**
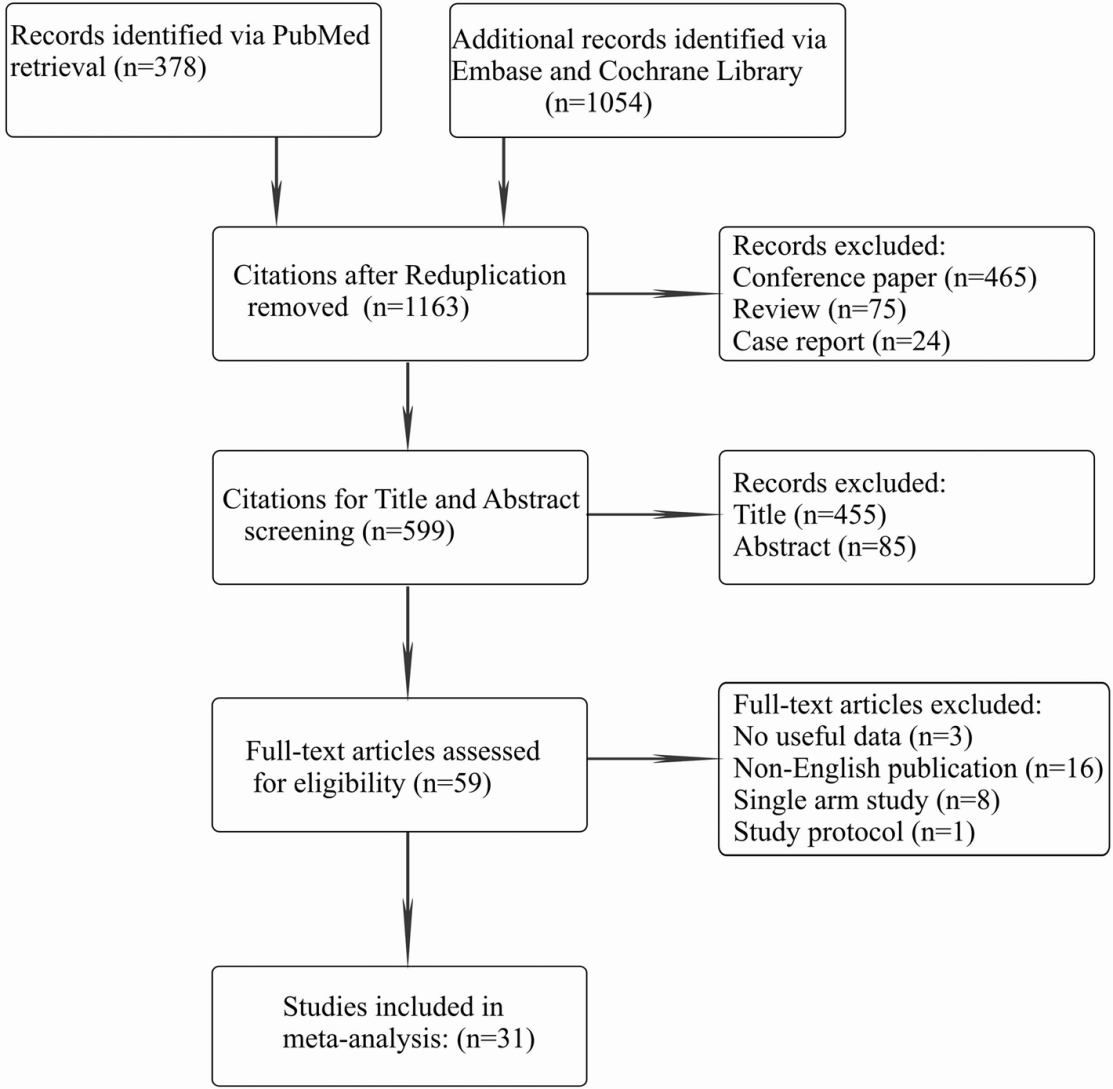
PRISMA flow diagram of the study selection

One thousand four hundred thirty-two potential citations were identified after systematic retrieval in the aforementioned databases. After the removal of duplicate citations (*N* = 269) and those types of work classified as review (*N* = 75), case report (*N* = 24) and conference paper (*N* = 465), 599 records were qualified for title and abstract screening; 211 of them were excluded by this process, leaving 59 articles for full-text evaluation. Of those, 28 were omitted for lack of useful data (*N* = 3), non-English publication (*N* = 16), single-arm study (*N* = 8) and study protocol (*N* = 1). Ultimately, 31 eligible trials [12, 14, 18–46] with 5125 unique patients with advanced HCC met the inclusion criteria. The PRISMA flow diagram of study selection is outlined in Fig. 1.

### Characteristics of included studies

Table 1 provides the details of the 31 included studies, and Table 2 summarises the characteristics of these studies in the “patient-level” analysis. Of those, six (19.4%) were RCTs that included a total of 1128 cases; 18 (58.1%) originated in China, and 15 (48.4%) applied 3–8-week frequency of tumour assessment, and the predominant treatment scenario was administration of 400 mg sorafenib orally twice a day. We also summarised other details in Tables 1 and 2, such as the publication year, median follow-up, median age in each treatment strategy, the primary endpoint and the chemotherapy drugs used in TACE.

**Table 1:** Characteristics of the eligible studies

Study	Study type	Original nation	Period	Follow-up frequency, weeks	No. of patients	Median age, years^a^	Follow-up, months^b^	Medication in TACE	Initial sorafenib administration	Trail	
Hoffmann et al. [24]	RCT	Germany	NR	NR	Combination, 24	58.5	33	Carboplatin	400 mg bid	24	
					TACE, 26	58.0					
Kudo et al. [26]	RCT	Japan	2006–2009	8	Combination, 138	69	NR	NR	400 mg bid	26	
					TACE, 187	69					
Lencioni et al. [27]	RCT	USA	NR	8	Combination, 154	64.5	9	DEB-TACE	400 mg bid	27	
					TACE, 153	63					
Lee et al. [29]	RCT	China, Taiwan	2009–2010	4–8	Combination, 36	62.3	NR	NR	400 mg bid	29	
					TACE, 36	62.6					
Sansonno et al. [30]	RCT	Italy	2007–2011	2–4	Combination, 31	73	NR	Doxorubicin-based	400 mg bid	30	
					TACE, 31	72.8					
Meyer et al. [35]	RCT	UK	2010–2015	6–12	Combination, 157	65	20.7	DEB-TACE	400 mg bid	35	
					TACE, 156	68					
Hu et al. [12]	Retrospective	China	2009–2013	6–8	Combination, 82	NR	6.9	Cisplatin-based	400 mg bid	12	
					TACE, 164						
Zhu et al. [14]	Retrospective	China	2010–2012	4–6	Combination, 46	48.4	11.3	Doxorubicin-based	400 mg bid	14	
					TACE, 45	51.9					
Qu et al. [18]	Retrospective	China	2008–2011	6–8	Combination, 45	51	NR	Epirubicin-based	200 mg bid	18	
					TACE, 45	49					
Wu et al. [19]	Retrospective	China	2004–2014	4–8	Combination, 56	47.6	NR	Doxorubicin-based	400 mg bid	19	
					Sorafenib, 48	50.2					
Tan et al. [20]	Retrospective	China	2004–2009	4–8	Combination, 10	46.3	NR	NR	400 mg bid	20	
					TACE, 10	43.4					
Bai et al. [21]	Retrospective	China	2004–2009	6	Combination, 82	54	1	Doxorubicin-based	400 mg bid	21	
					TACE, 146	52					
Lee et al. [22]	Retrospective	Korea	2000–2011	6–12	TACE, 26	58.3	NR	NR	NR	22	
					Sorafenib, 52	57.3					
Nishikawa et al. [23]	Retrospective	Japan	2004–2011	8–12	TACE, 55	67.9	NR	Epirubicin-based	200 mg bid	23	
					Sorafenib, 56	69.1					
Ren et al. [25]	Retrospective	China	2008–2015	6–8	Combination, 61	NR	NR	Oxaliplatin-based	400 mg bid	25	
					TACE, 247						
Arizumi et al. [28]	Retrospective	Japan	2008–2013	4–16	Combination, 32	73	NR	Epirubicin-based	400 mg bid	28	
					TACE, 24	77					
Ha et al. [31]	Retrospective	Korea	2007–2010	4–6	Combination, 129	54.1	NR	NR	400 mg bid	31	
					Sorafenib, 293	55.9					
Wan et al. [32]	Retrospective	China	2007–2011	4–12	Combination, 245	NR	35.8	Epirubicin-based	400 mg bid	32	
					TACE, 245						
Yao et al. [33]	Retrospective	China	2009–2015	4–6	Combination, 19	45.32	2-56	NR	400 mg bid	33	
					TACE, 78	46.67					
Wu et al. [34]	Retrospective	China	2009–2014	4–6	Combination, 30	NR	11.3	NR	400 mg bid	34	
					TACE, 31						
Ogasawara et al. [36]	Retrospective	Japan	2002–2011	8–16	Combination, 36	71	12.4	Epirubicin-based	400 mg bid	36	
					TACE, 20						
Yao et al. [37]	Retrospective	China	2011–2014	4–6	Combination, 50	56.5	13.9	Epirubicin-based	400 mg bid	37	
					TACE, 100	55.9					
Zhao et al. [38]	Retrospective	China	2009–2012	NR	Combination, 202	53	15.1	Doxorubicin-based	400 mg bid	38	
					TACE, 404	56					
Varghese et al. [39]	Retrospective	India	2010–2014	12–16	Combination, 37		NR	7	Doxorubicin-based	200 mg bid	39
					Sorafenib, 28						
Zhu et al. [40]	Retrospective	China	2010–2014	12	Combination, 40		55.5	63.0	Doxorubicin-based	400 mg bid	40
					TACE, 66		54.1				
Peng et al. [41]	Retrospective	China	2010–2015	4–8	Combination, 106		56.5	15.6	Epirubicin-based	400 mg bid	41
					Sorafenib, 101		56.3				
Pinter et al. [42]	Retrospective	Austria	1999–2009	8–16	TACE, 34		NR	8.0	DEB-TACE	400 mg bid	42
					Sorafenib, 63						
Zhang et al. [43]	Retrospective	China	2009–2013	4–8	Combination, 45		50.1	7.3	Epirubicin-based	400 mg bid	43
					Sorafenib, 44		53.6				
Lei et al. [44]	Retrospective	China	2009–2011	NR	Combination, 38		52	23	Oxaliplatin-based	400 mg bid	44
					TACE, 29		51				
Zheng et al. [45]	Retrospective	China	2008–2013	NR	Combination, 12		53	12.7	NR	200 mg bid	45
					TACE, 10						
Muhammad et al. [46]	Retrospective	USA	2007–2011	NR	Combination, 13		61.4	23	DEB-TACE	200 mg bid	46
					TACE, 30		59.2				

*Abbreviation*s: *NR* not reported in the text, *RCT* randomised controlled trial, *OS* overall survival, *TTP* time to progression, *ORR* overall response rate, *DPR* disease progression rate, *TACE* transarterial chemoembolization, *DEB-TACE* drug-eluting beads transarterial chemoembolization

^a^Sign indicates mean; otherwise, data are expressed as median

^b^Sign indicates median

**Table 2:** Summary of the characteristics of the 31 included studies in the “patient-level” analysis

Characteristic	Studies, no. (%) (***N*** = 31)	Advanced HCC patients, no. (%) (***N*** = 5125)
Study type		
RCT	6 (19.4)	1128 (22.0)
Retrospective	25 (80.6)	3997 (78.0)
Publication year, median (range)	2016 (2010–2019)	–
Follow-up, median (range), months	14.45 (5.4–63)	–
Median age, median (range), years		
Combination	56 (45.3–74)	–
TACE	57.5 (43.4–74)	–
Sorafenib	56.3 (50.2–74)	–
Original nation		
China	18 (58.1)	3082 (60.1)
USA	2 (6.5)	350 (6.8)
Japan	4 (12.9)	547 (10.7)
Korea	2 (6.5)	500 (9.8)
Germany	1 (3.2)	50 (1.0)
Italy	1 (3.2)	62 (1.2)
India	1 (3.2)	124 (2.4)
UK	1 (3.2)	313 (6.1)
Austria	1 (3.2)	97 (1.9)
Primary endpoint		
Overall survival	18 (58.1)	2431 (47.4)
Time-to-progression	4 (12.9)	743 (14.5)
Overall response rate	9 (29.0)	1508 (29.4)
Follow-up frequency		
3–8 weeks	15 (48.4)	2384 (46.5)
8–12 weeks	3 (9.7)	407 (7.9)
≥ 12 weeks	8 (25.8)	1249 (24.4)
Not assessed	5 (16.1)	1085 (21.2)
Medication in TACE		
Doxorubicin-based	7 (22.6)	1339 (26.1)
Epirubicin-based	8 (25.8)	856 (16.7)
DEB-TACE^b^	4 (12.9)	760 (14.8)
Others	4 (12.9)	1111 (21.7)
Not assessed	8 (25.8)	1059 (20.7)
Initial sorafenib administration		
200 mg bid	5 (16.1)	400 (7.8)
400 mg bid	25 (80.6)	4647 (90.7)
Not applicable	1 (3.2)	78 (1.5)

*Abbreviation*s: *HCC* hepatocellular carcinoma, *RCT* randomised controlled trial, *TACE* transarterial chemoembolization

^b^TACE with drug-eluting beads is performed with doxorubicin-loaded beads

### Time to progression and overall survival

**Fig. 2:**
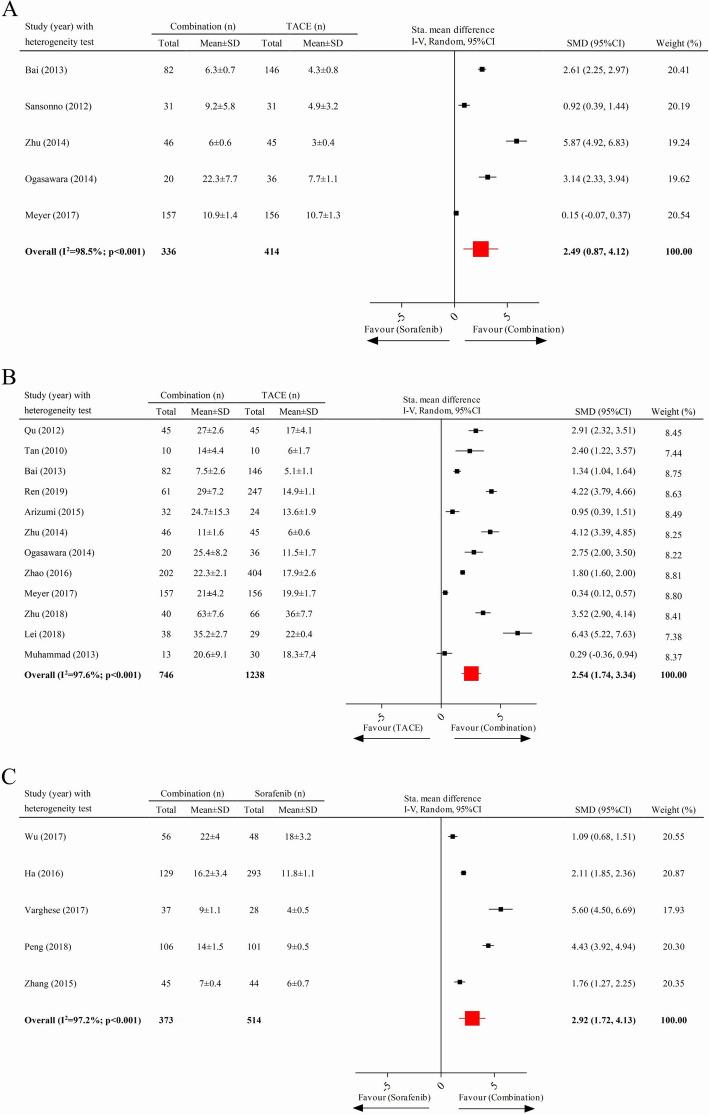
Comparison of time to progression and overall survival. **a** Time to progression between combination therapy and TACE. **b** Overall survival between combination and TACE. **c** Overall survival between combination and sorafenib

Five studies, containing 750 cases, were included in the analysis comparing the TTP with combination therapy to that of TACE; the pooled data showed that the TTP in patients with advanced HCC receiving combination therapy was significantly longer than that of those receiving TACE treatment alone (pooled SMD = 2.49; 95% CI 0.87–4.12) (Fig. 2a). Twelve clinical trials with 1984 cases and five available studies with 887 cases were respectively involved in the comparison of OS between combination therapy and TACE and that between combined therapy and sorafenib monotherapy. As presented in Fig. 2b and c, the combination therapy significantly prolonged the OS of patients compared to the monotherapy of TACE (pooled SMD = 2.54; 95% CI 1.74–3.34) or sorafenib (pooled SMD = 2.92; 95% CI 1.72–4.13).

### Disease progression rate

**Fig. 3:**
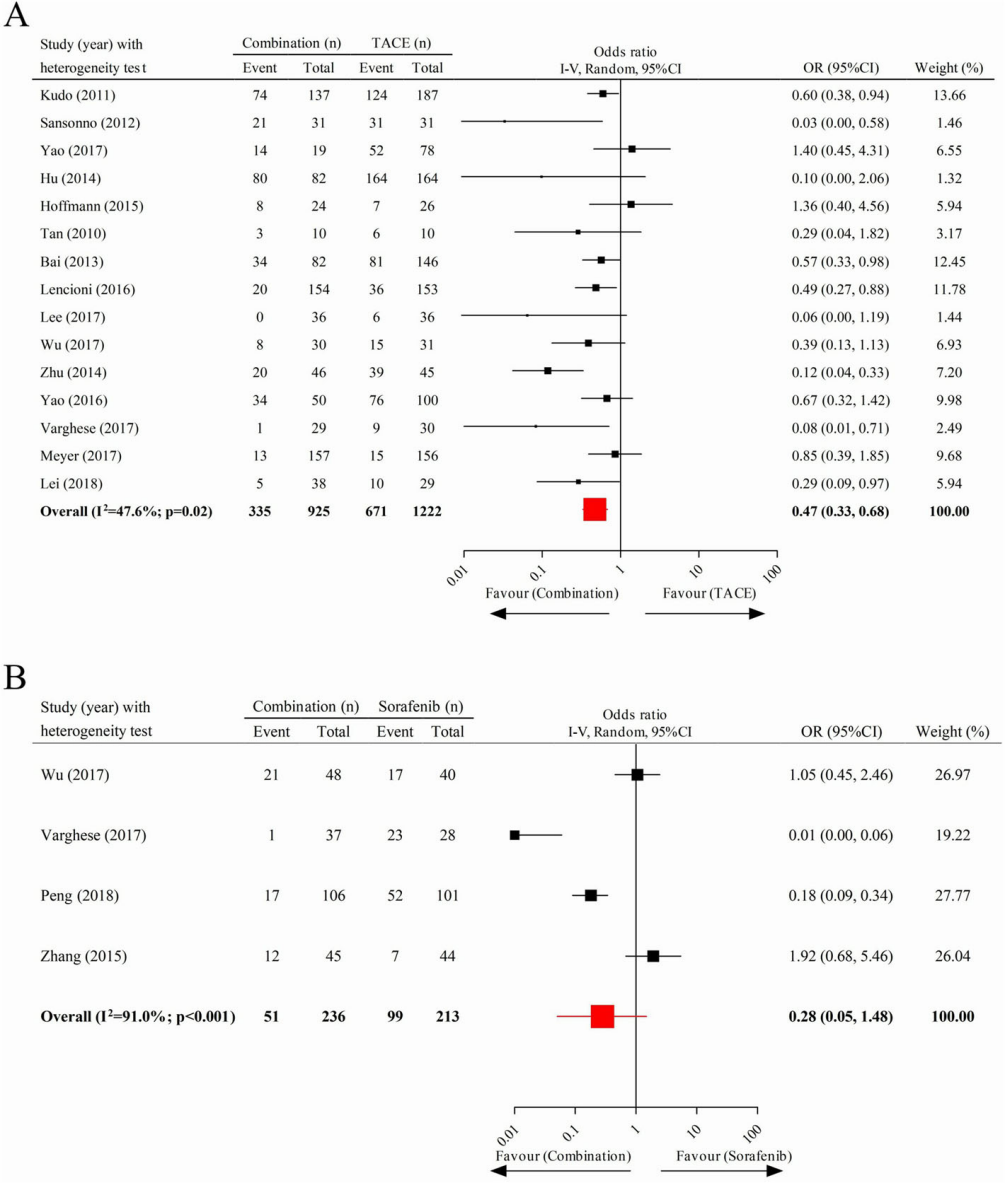
Comparison of disease progression rate. **a** Combination therapy vs. TACE. **b** Combination therapy vs. sorafenib

We obtained 15 and four articles, respectively, to compare the DPR under combined treatment to TACE and that of combination therapy to sorafenib. The pooled data indicated that patients with advanced HCC undergoing combination therapy had a significantly lower DPR than those who received monotherapy of TACE (pooled OR = 0.47; 95% CI 0.33–0.68) but not than those who took sorafenib alone orally (pooled OR = 0.28; 95% CI 0.05–1.48) (Fig. 3a, b).

### One-year and 2-year survival rates

**Fig. 4:**
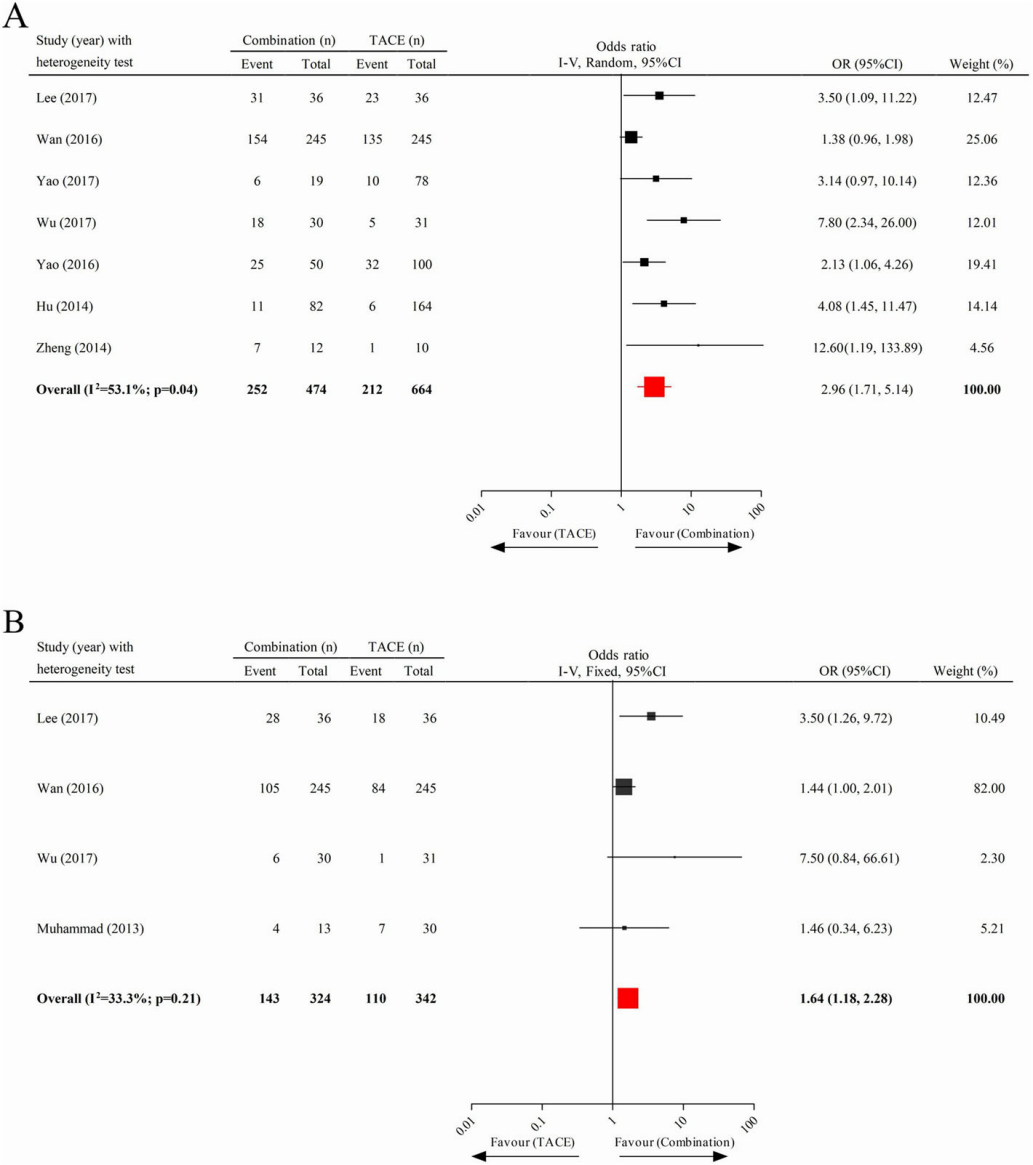
Analysis of the survival rate between the combination therapy cohort and the TACE cohort. **a** One-year survival rate. **b** Two-year survival rate

As shown in Fig. 4a and b, seven and four useful studies, respectively, were included to assess the 1-year and 2-year survival rates between combined treatment and TACE. Generally, patients in the combination therapy cohort benefited from significantly greater 1-year (pooled OR = 2.96; 95% CI 1.71–5.14) and 2-year (pooled OR = 1.64; 95% CI 1.18–2.28) survival rates than those in the TACE monotherapy cohort. We further analysed the survival rates of patients who received monotherapy with TACE compared to sorafenib, finding no significant difference of 1-year (pooled OR = 1.39; 95% CI 0.84–2.29) and 2-year (pooled OR = 1.70; 95% CI 0.69–4.18) survival rates between them (eFigure 1 in Supplementary page 1).

### Overall response rate

**Fig. 5:**
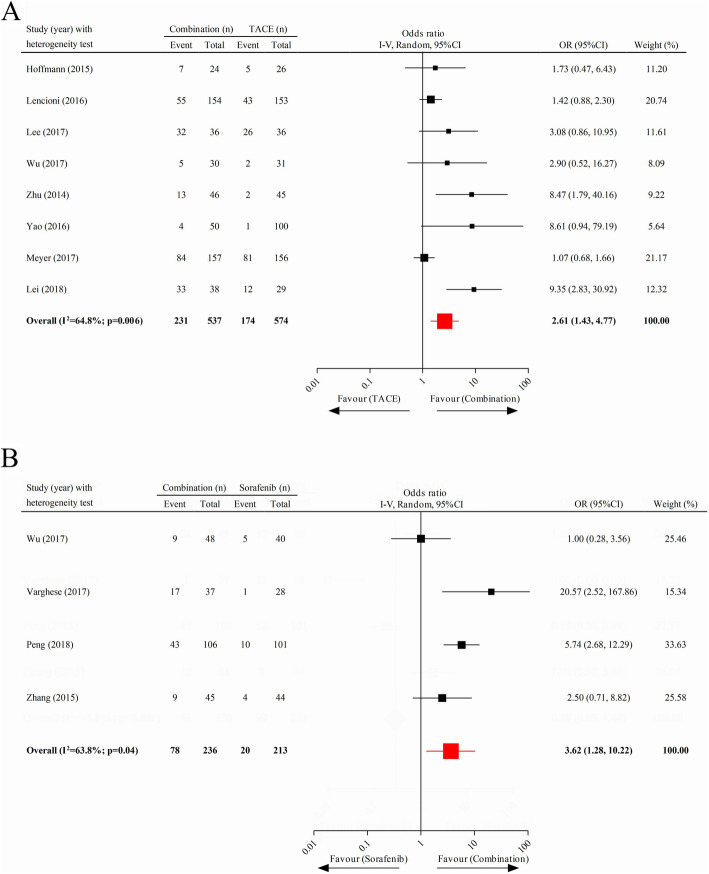
Comparison of overall response rate. **a** Combination therapy versus TACE. **b** Combination therapy versus sorafenib

Eight and four studies, respectively, were involved in the comparison of ORR between combination therapy and TACE and that between combination therapy and sorafenib. The results of the analysis suggested that patients exhibited a significantly increased ORR after receiving combination therapy, compared to those who underwent monotherapy with TACE (pooled OR = 2.61; 95% CI 1.43–4.77) or sorafenib (pooled OR = 3.62; 95% CI 1.28–10.22) (Fig. 5a, b).

### Publication bias

The publication bias tests in most analyses were devoid of statistical significance, indicating no occurrence of publication bias among the studies involved in them; however, the analyses of DPR, 1-year survival rate and ORR after combination therapy versus TACE manifested discernible publication bias (*P* = 0.04,0.00 and 0.01, respectively) (eTable 1 on Supplementary page 2).

## Discussion

Universally, patients with advanced HCC suffered from a poor prognosis due to the lack of surgical resection opportunity and sustained their survival only with some palliative treatments. This meta-analysis proves that the combination therapy of TACE and sorafenib provides more advantages to improve ORR and prolong OS than monotherapy with either in treating these patients and enhances the increased 1-year and 2-year survival rates, lengthens TTP and decreases DPR, compared to TACE alone. We further found equivalent 1-year and 2-year survival rates between those who received TACE and only orally took sorafenib.

Several meta-analyses [47–50] and one case-control matched study [51] have reached a consensus that there is a significant improvement of TTP with a combined treatment arm, compared to a TACE-alone arm, but discordance exists with reference to the OS outcomes between them. Of those studies, some results indicated that the use of sorafenib in patients with HCC concomitantly receiving TACE did not ameliorate the OS compared to those only receiving TACE alone [47–49], whereas Yang et al. [50] pointed out that the OS results favoured the combined-treatment group rather than the TACE monotherapy group. The inconsistent conclusions may be because the study by Yang et al. explicitly confines the inclusion criteria to unresectable HCC patients, whereas others did not stipulate this limitation and had fewer analysed subjects. Similarly, two retrospective studies [52, 53] also reported a favourable OS in the combination group as compared to TACE alone.

A systematic review divided patients with advanced HCC based on their region into two subgroups: an Asian countries group and a Western countries group and, interestingly, revealed that the TTP and OS were exclusively prolonged in the Asian countries group but not in the Western countries group after combination therapy, suggesting that the efficacy of combined treatment might be affected by race [54]. Previous meta-analysis showed more improvement in 0.5-year and 1-year survival rates of patients with advanced HCC who underwent combined therapy than those who underwent TACE monotherapy [55]; consistently, our results further support this study, affirming that the 2-year survival rate of those patients was also increased by sorafenib in combination with TACE, even without the diversity of 1-year and 2-year survival rates between the TACE monotherapy cohort and the sorafenib-alone cohort.

Our result, that the OS of patients with advanced HCC treated with combination therapy was superior to that of those patients treated with sorafenib alone, maps to the conclusions of 4 retrospective studies [19, 31, 56, 57] but is in contrast to a clinical trial by Zhang and colleagues in 2015 [43]. In this study, despite a numerically greater median OS in the combined-therapy group than in the sorafenib-alone group (7.3 months vs. 6.0 months), no difference was observed between the two groups (*P* = 0.924). The contradiction may be because the enrolled patients in the study by Zhang et al. concurrently had main portal vein tumour thrombosis, which may be an unfavourable factor that affected the efficacy of the combined therapy [14].

Our results mirror the findings from two meta-analyses in which both corroborate the improved tumour regression and disease control of patients with advanced HCC after combination therapy compared to TACE monotherapy [55, 58]; however, the combined treatment may not be superior to TACE alone to increase the ORR and curtail the DPR in patients with early-to-intermediate HCC [59]. In this present analysis, we moreover demonstrate the better ORR in the combination therapy arm than that in the sorafenib-alone arm, but the conclusions of three key clinical trials in this context are contradictory [19, 39, 60]. One explanation is that one of them classified patients with advanced HCC into a BCLC-B stage group and a BCLC-C stage group and demonstrated that the superiority of combination therapy compared to sorafenib monotherapy was manifested only in the former group but not in the latter group, whereas the other studies did not implement this subgroup analysis.

The DPR in the combined-treatment cohort is not greater than that in the sorafenib monotherapy cohort, which may be consistent with the outright opposite effects of expressing the hypoxia-inducible factor-1α (HIF-1α) and VEGF in patients with advanced HCC undergoing TACE and in those after treatment with sorafenib. First, tumour-feeding arteries are embolised by TACE treatment, inevitably giving rise to the elevation of the HIF-1α level that is related to tumour recurrence, disease progression and distant metastasis [21, 61]. Second, TACE incites the overexpression of VEGF in HCC, hence promoting angiogenesis [6, 7]. By contrast, sorafenib effectively reduces the expression of HIF-1α and VEGF and inhibits VEGF receptor and platelet-derived growth factor receptors, which alleviates the TACE-induced adverse situations [62–64]. Therefore, TACE in conjunction with sorafenib to treat patients with advanced HCC theoretically does not outperform sorafenib alone in decreasing the DPR.

The study has some limitations that deserve special mention. First, including only English language articles might lead to selection bias. Furthermore, only several trials with limited available data were enrolled to conduct some analyses (the DPR of combination therapy versus sorafenib, the 2-year survival rate of combination therapy versus TACE and the ORR of combination therapy versus sorafenib), which might increase the uncertainty of the conclusions. Third, substantial heterogeneity was manifested in almost all analyses, which may be relevant to the differences of study type, treatment procedures and the frequency of tumour assessment. The *P* value of Egger’s test in some analyses also suggested potential publication bias. Additionally, treatment-related adverse events were not assessed in our article because they were tolerable [55]. Last, hepatitis B (HB) virus infection accounts for the predominant reason for HCC, particularly in China, and anti-HB virus therapy can significantly ameliorate HCC patients who house HB virus; however, there were scanty details documented in these included clinical trials. If it is available to implement a stratified analysis of HCC patients with or without HB infection, there may be some innovated results.

## Conclusion

The combination of TACE with sorafenib in treating patients with advanced HCC can prolong TTP and OS, improve ORR and 1-year and 2-year survival rates and reduce the DPR more efficiently than TACE can alone. This combination therapy is also superior to sorafenib monotherapy in terms of the longer OS and higher ORR. As a monotherapy strategy, the 1-year and 2-year survival rates in the TACE arm were identical to those in the sorafenib arm.

## Supplementary information

**Additional file 1: eFigure 1**. The comparison of survival rate between TACE and sorafenib. (A) 1-year survival rate; (B) 2-year survival rate. **eTable 1**. The publication bias in all analyses. Abbreviations: TTP, time-to-progression; OS, overall survival; DPR, disease progression rate; 1y-SR, 1-year survival rate; 2y-SR, 2-year survival rate; ORR, overall response; TACE, transarterial chemoembolization.

## Data Availability

All data are fully available without restriction.

## Abbreviations

HCC: Hepatocellular carcinoma
TACE: Transarterial chemoembolisation
OR: Odds ratio
CI: Confidence interval
SMD: Standardised mean difference
OS: Overall survival
TTP: Time to progression
ORR: Overall response rate
DPR: Disease progression rate
BCLC: Barcelona Clinic Liver Cancer
VEGF: Vascular endothelial growth factor
RECIST: Response Evaluation Criteria in Solid Tumours
mRECIST: Modified Response Evaluation Criteria in Solid Tumours
RCTs: Randomised controlled trials
HIF-1α: Hypoxia-inducible factor-1α
