# Supplementary material for: The combination therapy of transarterial chemoembolisation and sorafenib is the preferred palliative treatment for advanced hepatocellular carcinoma patients: a meta-analysis
Source: World J Surg Oncol. 2020 Sep 11;18:243. doi: 10.1186/s12957-020-02017-0 (PMC7488414; doi:10.1186/s12957-020-02017-0)
Supplement: Supplementary file 1 — Additional file 1: eFigure 1. The comparison of survival rate between TACE and sorafenib. (A) 1-year survival rate; (B) 2-year survival rate. eTable 1. The publication bias in all analyses. Abbreviations: TTP, time-to-progression; OS, overall survival; DPR, disease progression rate; 1y-SR, 1-year survival rate; 2y-SR, 2-year survival rate; ORR, overall response; TACE, transarterial chemoembolization. [file 12957_2020_2017_MOESM1_ESM.docx]

**eFigure 1: The comparison of survival rate between TACE and sorafenib.**

A: 1-year survival rate;

B: 2-year survival rate.


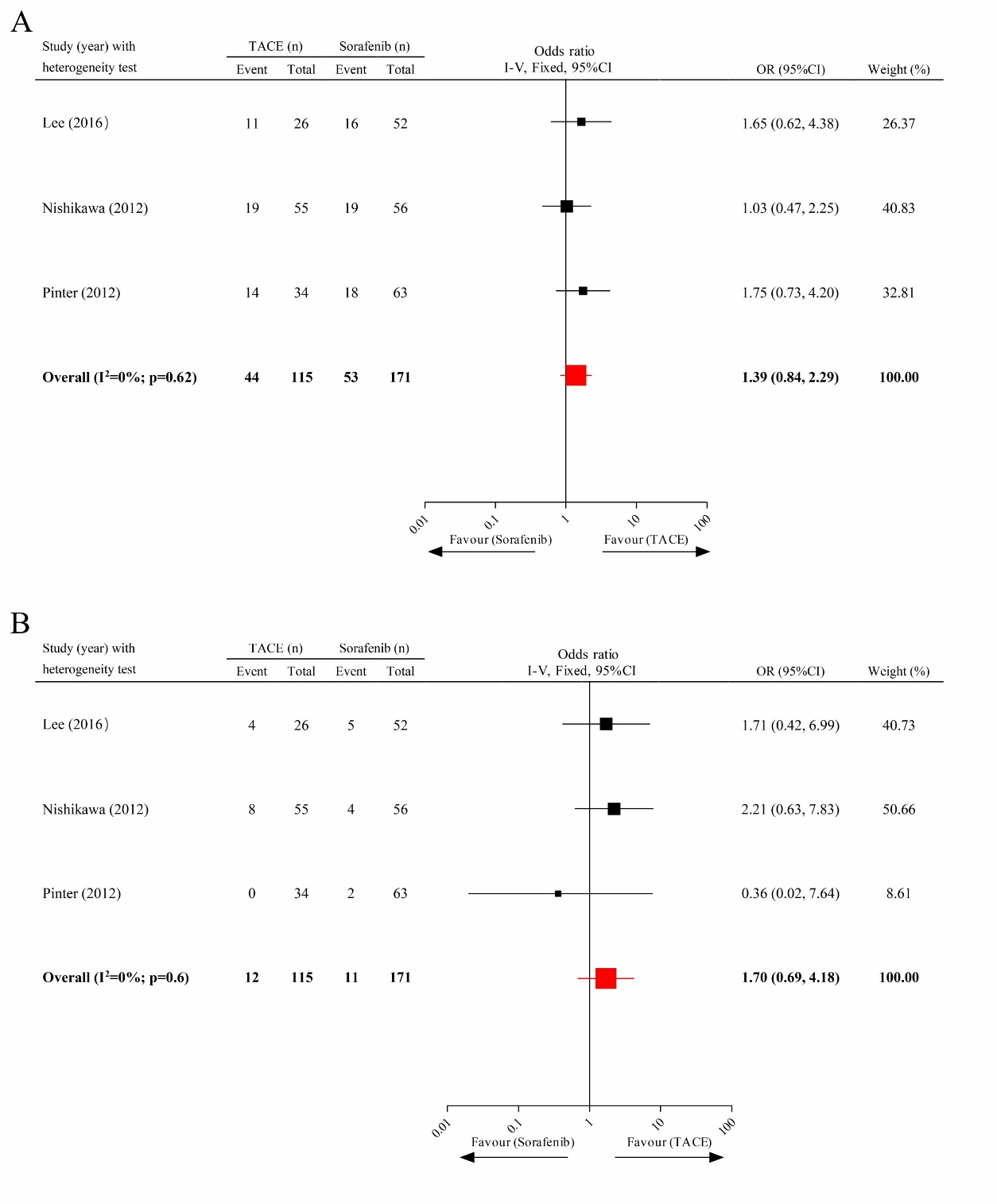


| **Analysis label** | **P value** | **Publication bias** |
| --- | --- | --- |
| TTP in Combination vs TACE | 0.10 | No |
| OS in Combination vs TACE | 0.07 | No |
| OS in Combination vs Sorafenib | 0.35 | No |
| DPR in Combination vs TACE | 0.04 | Yes |
| DPR in Combination vs Sorafenib | 0.76 | No |
| 1y-SR in Combination vs TACE | 0.00 | Yes |
| 2y-SR in Combination vs TACE | 0.23 | No |
| 1y-SR in TACE vs Sorafenib | 0.39 | No |
| 2y-SR in TACE vs Sorafenib | 0.07 | No |
| ORR in Combination vs TACE | 0.01 | Yes |
| ORR in Combination vs Sorafenib | 0.95 | No |

**eTable 1. The publication bias in all analyses.**

Abbreviations: TTP, time-to-progression; OS, overall survival; DPR, disease progression rate; 1y-SR, 1-year survival rate; 2y-SR, 2-year survival rate; ORR, overall response; TACE, transarterial chemoembolization.
